# In Vivo Ergogenic Properties of the *Bifidobacterium longum* OLP-01 Isolated from a Weightlifting Gold Medalist

**DOI:** 10.3390/nu11092003

**Published:** 2019-08-24

**Authors:** Mon-Chien Lee, Yi-Ju Hsu, Hsiao-Li Chuang, Pei-Shan Hsieh, Hsieh-Hsun Ho, Wei-Ling Chen, Yen-Shuo Chiu, Chi-Chang Huang

**Affiliations:** 1 Graduate Institute of Sports Science, National Taiwan Sport University, Taoyuan City 33301, Taiwan; 2 National Laboratory Animal Center, National Applied Research Laboratories, Taipei 11529, Taiwan; 3 Glac Biotech Co., Ltd., Tainan City 74442, Taiwan; 4 Department of Sports Training Science-Athletics, National Taiwan Sport University, Taoyuan City 33301, Taiwan; 5 Department of Orthopedic Surgery, Taipei Medical University Shuang Ho Hospital, New Taipei City 23561, Taiwan

**Keywords:** olympics, OLP-01, gold medal, probiotic, exercise performance, weightlifting

## Abstract

In recent years, probiotics of human origin have shown superior results and performance compared to probiotics from plant or dairy sources, in both in vitro and animal studies. Towards this end, the current study was conducted to explore the ergogenic properties of *Bifidobacterium longum subsp. longum* OLP-01 isolated from the intestinal microbiome of the gold medalist from the 2008 Beijing Olympics women’s 48 kg weightlifting competition. Male Institute of Cancer Research (ICR) mice were divided into four groups (*n* = 10 per group) and orally administered OLP-01 for 4 weeks at 0 (vehicle), 2.05 × 10^9^ (OLP-01-1X), 4.10 × 10^9^ (OLP-01-2X), and 1.03 × 10^10^ (OLP-01-5X) CFU/kg/day. Physical performance tests including grip strength and endurance time were measured, with OLP-01 supplementation dose-dependently elevating grip strength and endurance. The anti-fatigue activity levels of serum lactate, ammonia, glucose, blood urea nitrogen (BUN), and creatine kinase (CK) were measured after an acute exercise challenge, and OLP-01 was found to significantly decrease lactate, ammonia, and CK levels. OLP-01 treatment was also found to significantly increase the resting levels of both hepatic and muscular glycogen, an indicator of energy storage. Supplementation by OLP-01 showed no subchronic toxic effects while supporting many health-promoting, performance-improving, and fatigue-ameliorating functions.

## 1. Introduction

Exercise increases the alpha diversity of the microbiome of individuals and decreases the thick-walled bacteria, and *Clostridium* is considered to be one of the main factors regulating the composition and health of intestinal microbial diversity [1]. Exercise promotes the energy harvesting functions of gut microbiota such as antibiotic biosynthesis, amino acid utilization, carbohydrate metabolism, and production of fecal metabolites, e.g., short-chain fatty acids (SCFAs). This leads to enhanced muscle turnover, adaptation, and improved exercise performance. [2]. Athletes have been reported to have a higher diversity of gut microbes, representing 22 distinct phyla, compared to non-athletes with BMI < 25 or BMI > 28 [3]. Exercise can alter the gut microbiota, but there have been limited studies showing that athletic performance may be improved by increasing the beneficial microbe in the gut.

The modern definition of probiotic was put forward as an individual or mixed culture of bacteria which, when applied to an animal or human, affects the host beneficially by shaping the indigenous microbiota [4]. There have been many previous studies showing that supplementation of probiotics is able to exert anti-inflammatory properties [5], reduce the incidence of obesity and its related metabolic diseases [6], improve Type 2 diabetes mellitus [7], modulate hypocholesterolemic properties [8], and promote antioxidant responses [9]. There are specific probiotic strains with demonstrated health benefits from the following genera: *Lactobacillus, Bifidobacterium, Streptococcus, Pediococcus, Saccharomyces, Bacillus, Leuconostoc, Enterococcus*, and *Escherichia coli* [10]. Most probiotics come mainly from fermented food such as yogurt, fermented milk, cheese, kimchi, granola bar, microalgae, and miso [11]. In recent years, the development and application of human-origin probiotics has increasingly received attention as many studies have shown their superior results and performance in both in vitro and animal studies, compared to probiotics from plant and dairy sources. This appears to be the current trend in the development of probiotics [12,13].

*Bifidobacterium longum* is a rod-shaped, Gram-positive and catalase-negative bacterium, commonly inhabiting the intestinal tract of humans [14]. *B. longum*, along with other Bifidobacterium species, colonizes the human gastrointestinal (GI) tract, where it represents up to 90% of the bacteria of an infant’s GI tract [15]. After weaning, this number gradually reduces to 3% in an adult’s GI, and Bacteroides and Eubacterium begin to dominate with age [16]. There have been few previous studies looking at the supplementation of *B. longum*. Most of these studies have looked at the effect of *B. longum* mixed with other strains, and have shown improvement in cognitive function [17] and muscular performance [18], reduced obesity [19], and amelioration of colitis and liver injuries [20]. Studies looking at its application and benefit in sport sciences, however, have been limited. Recently, a specific probiotic strain termed *B. longum* (OLP-01) was isolated from the 2008 Olympic women’s 48 kg weightlifting gold medalist’s gut microbiota. We hypothesized that OLP-01 contributes to host energy utilization and mediates the host’s characteristic properties in terms of exercise performance, physical fatigue, and body composition. We tested different doses of OLP-01 supplementation on physical performance in vivo, and we then evaluated anti-fatigue function in OLP-01-treated mice.

## 2. Materials and Methods

### 2.1. OLP-01 Preparation

*Bifidobacterium longum subsp. Longum* OLP-01 was isolated from Wei-Ling Chen, gold medal winner of the 2008 Summer Olympics women’s 48 kg weightlifting competition. The bacterial strain was confirmed by an independent third party, the Food Industry Research and Development Institute (Hsinchu, Taiwan). The dry product of OLP-01 was prepared and provided by Glac Biotech Co., Ltd. (Tainan, Taiwan), and the viable cell count of OLP-01 was 1.07 × 10^11^ CFU/g. Before supplementation, cells were suspended in phosphate buffered saline (PBS), pH 7.2.

### 2.2. Animals and Experimental Design

Male 7 week old ICR mice under specific pathogen-free conditions were purchased from BioLASCO (Yi-Lan, Taiwan). All animals were provided with distilled water and standard chow diet (No. 5001; PMI Nutrition International, Brentwood, MO, USA) ad libitum, and maintained at a regular cycle (12 h light/dark) at room temperature (23 ± 2 °C) and 50–60% humidity. All animal experiments in this study conformed to the guidelines of protocol IACUC-10717 and were approved by the Institutional Animal Care and Use Committee (IACUC) of National Taiwan Sport University.

The human dose of OLP-01, 1 × 10^10^ CFU per day was modified from previous studies [21]. The doses administered to the mice were converted from a human-equivalent dose (HED) based on body surface area, provided by the U.S. Food and Drug Administration and using the conversion coefficient of 12.3 for the mouse species. After a 1 week acclimation period, the 8 week old ICR mice were divided into four groups (*n* = 10 per group) and administered by oral gavage once daily for 4 weeks: (1) vehicle group (0 CFU/kg); (2) OLP-01-1X group (2.05 × 10^9^ CFU/kg); (3) OLP-01-2X group (4.10 × 10^9^ CFU/kg), and (4) OLP-01-5X group (1.03 × 10^10^ CFU/kg). All groups were administered the same volume of PBS or supplement and the OLP-01 dose determined according to the body weight of each mouse.

### 2.3. Forelimb Grip Strength

A low-force testing system (Model-RX-5, Aikoh Engineering, Nagoya, Japan) was used to measure the grip strength of mice undergoing vehicle or OLP-01 supplementation. The details have been described previously [22].

### 2.4. Exercise Endurance Test

The swim-to-exhaustion exercise test involved mice carrying constant loads corresponding to 5% body weight (BW) to analyze endurance time, as previously described [23]. The swimming endurance time of each mouse was recorded from beginning to exhaustion, which was determined by observing loss of coordinated movements and failure to return to the surface within 7 s [24].

### 2.5. Determination of Fatigue-Associated Biochemical Variables

The fatigue-related biochemical indicators selected to accurately demonstrate physiological status and assess the effects of OLP-01 supplementation were based on our previous reports [25]. The fatigue-associated variables were measured under fasting conditions to reflect the real physiological adaptation under exercise interventions. Blood samples were collected after 10 min of the swimming exercise and following 20 min of rest. Samples were centrifuged at 1500× *g* for 10 min at 4 °C and serum collected. Serum lactate, ammonia, and glucose levels were determined by an autoanalyzer (Hitachi 7060, Hitachi, Tokyo, Japan). The other variables, such as blood urine nitrogen (BUN) and creatine kinase (CK), were assessed immediately after 90 min extended exercise and 60 min of rest.

### 2.6. Resting Biochemical Profiles at the End of the Study

At the end of the study, all mice were euthanized by 95% CO_2_ asphyxiation one hour after the last treatment and blood obtained by cardiac puncture. Serum was collected after centrifugation and biochemical indexes assessed by Hitachi 7060 autoanalyzer. Levels of aspartate aminotransferase (AST), alanine transaminase (ALT), total cholesterol (TC), triglycerides (TG), creatine kinase (CK), glucose (GLU), albumin (ALB), blood urea nitrogen (BUN), creatinine (CREA), uric acid (UA), and total protein (TP) were measured.

### 2.7. Body Composition, Glycogen Content, and Histopathology

After the mice were euthanized, the liver, kidney, heart, lung, muscle, epididymal fat pad (EFP), and brown adipose tissue (BAT) were accurately excised and weighed. Organs were carefully removed, minced, and fixed in 10% formalin. Tissues were embedded in paraffin and cut into 4 μm thick slices for morphological and pathological evaluations. Sections were stained with hematoxylin and eosin (H & E) and examined under a light microscope equipped with a charge-coupled device (CCD) camera (BX-51, Olympus, Tokyo, Japan) by a clinical pathologist, as previously described [23]. Parts of the muscle and liver tissues were stored in liquid nitrogen for glycogen content analysis, as previously described [26].

### 2.8. Bacterial DNA Extraction and 16S rRNA Sequencing

According to the method previously used by our laboratory, the collected samples were immediately stored at −80 °C for DNA extraction after the mice were euthanized. A detailed procedure for sample extraction, preparation, and analysis has been described previously [22].

### 2.9. Statistical Analysis

All the statistical analysis was performed using SAS 9.4 (SAS Inst., Cary, NC, USA) and analyzed by one-way analysis of variance (ANOVA). The Cochran–Armitage test was used for the dose-effect trend analysis. Data have been expressed as mean ± SD with statistical significance set at *p* < 0.05.

## 3. Results

### 3.1. Effect of OLP-01 Supplementation on Body Weight, Food and Water Intake, and Organ Weights

**Figure 1:**
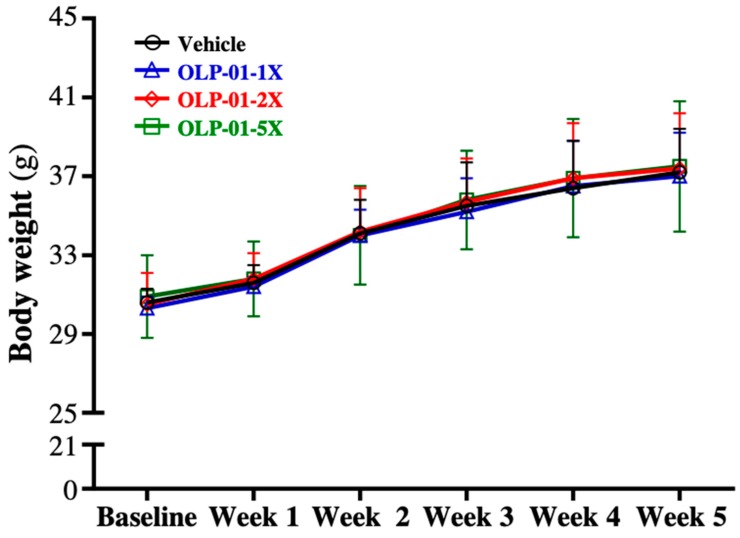
The effect of OLP-01 supplementation on growth curves. Data are expressed as mean ± SD for *n* = 10 mice per group.

The body weight, food intake, water intake, and body compositions are shown in Table 1. The results showed no significant differences in the 4 week growth curves of the vehicle, OLP-01-1X, OLP-01-2X, and OLP-01-5X groups (Figure 1). There was no significant difference in the body weight, food intake, water intake, or body composition among the groups. Only relative EFP weight was significantly lowered by 23.44% (*p* = 0.0376), 25.04% (*p* = 0.0255), and 23.60% (*p* = 0.0338) with 1X, 2X, and 5X OLP-01 supplementation, respectively, compared to the vehicle group.

### 3.2. Effect of OLP-01 Supplementation on Grip Strength

**Figure 2:**
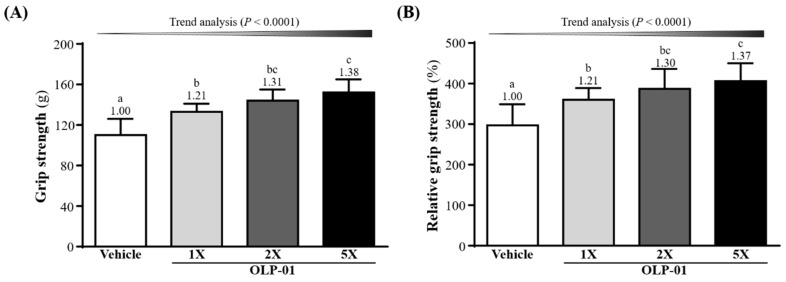
Effect of 4 week OLP-01 supplementation on (**A**) absolute forelimb grip strength and (**B**) forelimb grip strength (%) relative to body weight. Data are expressed as mean ± SD for *n* = 10 mice per group. Different superscript letters (a, b, c) indicate significant difference at *p* < 0.05.

As shown in Figure 2A, the mean forelimb grip strengths in the vehicle, OLP-01-1X, OLP-01-2X, and OLP-01-5X groups were 110 ± 16, 133 ± 8, 144 ± 11, and 152 ± 13 g, respectively. This represented a 1.21-fold (*p* = 0.0002), 1.31-fold (*p* < 0.0001), and 1.38-fold (*p* < 0.0001) increase in grip strength, respectively, compared with the vehicle group. Relative grip strength (%) as calculated by normalizing to individual body weight, was also significantly higher with OLP-01 treatment (Figure 2B). Both absolute and relative grip strength measurements showed significant OLP-01 dose-dependent effects (*p* < 0.0001) in trend analysis. Thus, OLP-01 supplementation for 4 weeks was able to improve forelimb grip strength.

### 3.3. Effect of OLP-01 Supplementation on Endurance Capacity in the Swim-to-ExhaustionTest

**Figure 3:**
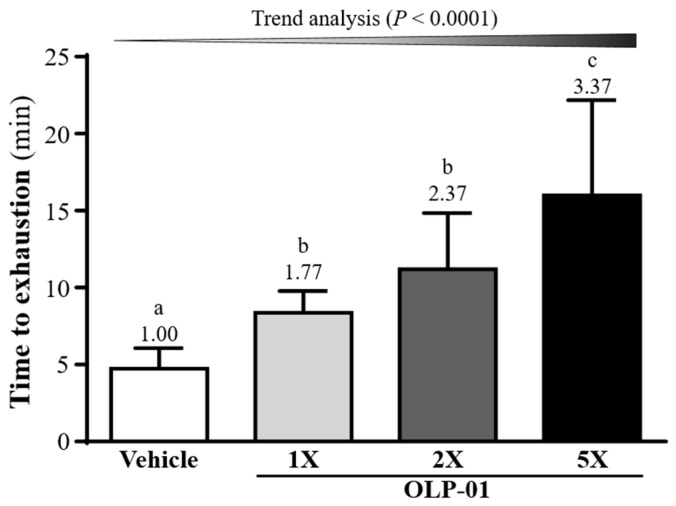
Effect of 4 weeks of OLP-01 supplementation on exhaustive swim times. Data are expressed as mean ± SD for *n* = 10 mice per group. Different superscript letters (a, b, c) indicate significant difference at *p* < 0.05.

In Figure 3, the exhaustive swim times in the vehicle, OLP-01-1X, OLP-01-2X, and OLP-01-5X groups were 4.74 ± 1.33, 8.36 ± 1.42, 11.20 ± 3.65, and 15.98 ± 6.20 min, respectively. This corresponded to a 1.77-fold (*p* = 0.0360), 2.37-fold (*p* = 0.0004), and 3.37-fold (*p* < 0.0001) increase, respectively, in the OLP-01-1X, OLP-01-2X, and OLP-01-5X groups, compared with the vehicle group. In the trend analysis, maximal swim time was increased dose-dependently with OLP-01 supplementation (*p* < 0.0001).

### 3.4. Effect of OLP-01 Supplementation on Lactate after the 10 Min Swim Test

After 4 weeks of supplementation, mice underwent a 10 min swim test to evaluate the levels of lactate, a metabolite highly associated with exercise physiological status. Lactate levels were assessed at three time points: pre-exercise, post- exercise, and after 20 min rest (Table 2). Before swimming and at baseline, there was no significant difference in the levels of blood lactate among the groups. After the 10 min swim test, the levels of blood lactate in vehicle, OLP-01-1X, OLP-01-2X, and OLP-01-5X groups were 6.74 ± 0.28, 5.64 ± 0.57, 5.40 ± 0.83, and 5.12 ± 0.59 mmol/L, respectively. This represented an increase of 16.38% (*p* = 0.0002), 19.82% (*p* < 0.0001), and 24.02% (*p* < 0.0001) in the OLP-01-1X, OLP-01-2X, and OLP-01-5X groups respectively, compared to the vehicle group. In addition, there was a significant dose-dependent effect on the post-exercise lactate levels (*p* < 0.0001).

After 20 min rest following the swimming test, the blood lactate levels in vehicle, OLP-01-1X, OLP-01-2X, and OLP-01-5X groups were 6.05 ± 0.25, 5.04 ± 0.65 4.76 ± 0.67, and 4.53 ± 0.57 mmol/L, respectively. Compared with the vehicle group, OLP-01-1X, OLP-01-2X, and OLP-01-5X were significantly lower than vehicle by 16.79% (*p* = 0.0003), 21.30% (*p* < 0.0001), and 25.14% (*p* < 0.0001). This decrease in blood lactate level after rest was shown in the trend analysis to be dose-dependent (*p* < 0.0001). Lactate production rates were significantly changed in all the OLP-01 supplementation groups (*p* < 0.0001) compared to the vehicle group, but lactate clearance rates were not significantly different among the groups.

### 3.5. Effect of OLP-01 Supplementation on Ammonia and Glucose after the 10 Min Swim Test

**Figure 4:**
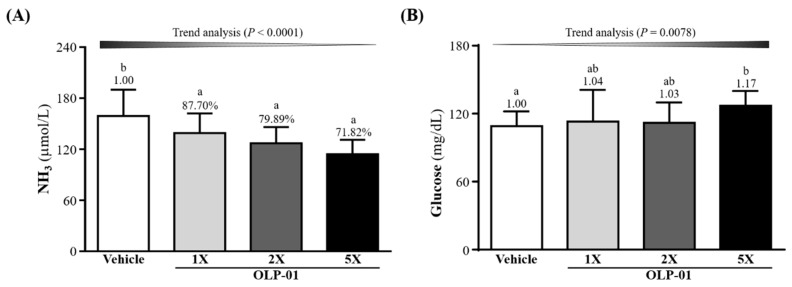
Effect of 4 week OLP-01 supplementation on serum (**A**) ammonia (NH_3_) and (**B**) glucose levels after 10 min acute exercise challenge. Data are expressed as mean ± SD for *n* = 10 mice per group and the different superscript letters (a, b) indicate significant difference at *p* < 0.05.

The serum ammonia levels in the mice were measured (Figure 4A) level were found to be 6.05 ± 0.25, 5.04 ± 0.65 4.76 ± 0.67, and 4.53 ± 0.57 μmol/L in the vehicle, OLP-01-1X, OLP-01-2X, and OLP-01-5X groups, respectively. The ammonia levels were 20.11% (*p* = 0.0044) and 28.18% (*p* = 0.0001) lower in the OLP-01-2X and OLP-01-5X groups compared to the vehicle group. A significant dose-dependent effect on blood ammonia levels was observed (*p* < 0.0001) after the 10 min swim test. Blood glucose levels were also obtained immediately after the 10 min swim test (Figure 4B). Only in the OLP-01-5X group was the blood glucose level significantly increased, 1.17-fold (*p* = 0.0411) compared with the vehicle group. However, trend analysis showed a significant dose-dependent effect (*p* = 0.0078) of OLP-01 supplementation on blood glucose levels.

### 3.6. Effect of OLP-01 Supplementation on BUN and CK after a 90 Min Swim Test and 60 Min Rest Period

**Figure 5:**
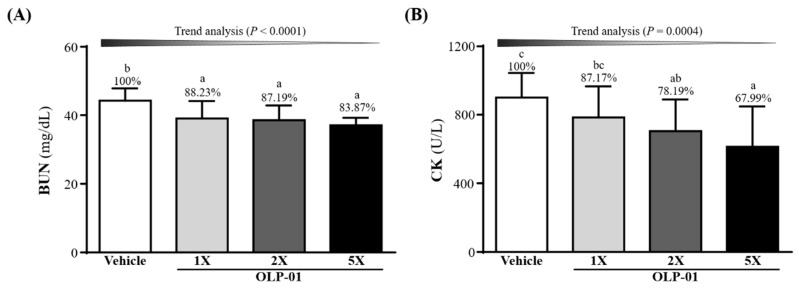
Effect of 4 week OLP-01 supplementation on serum (**A**) BUN and (**B**) CK levels after extended exercise challenge. The indicated four groups underwent 90 min swim exercise and blood was sampled after 60 min of rest. Data are expressed as mean ± SD for *n* = 10 mice per group and the different superscript letters (a, b, c) indicate significant difference at *p* < 0.05.

The mouse groups were subjected to an extended 90 min swim test followed by 60 min rest and measured for BUN levels (Figure 5A). OLP-01 supplementation (OLP-01-1X, OLP-01-2X, and OLP-01-5X) significantly decreased the exercise-induced BUN elevation by 11.77% (*p* = 0.0054), 12.81% (*p* = 0.0027), and 16.13% (*p* = 0.0003), respectively, showing a dose-dependent trend (*p* < 0.0001). The other important injury index, CK, also showed significant difference among the groups (Figure 5B). OLP-01-2X, and OLP-01-5X supplementation could significantly decrease the CK levels by 21.81% (*p* = 0.0264) and 32.01% (*p* = 0.0017) compared to the vehicle group, and showed a dose-dependent trend (*p* = 0.0004).

### 3.7. Effect of OLP-01 Supplementation on Liver and Muscle Glycogen

**Figure 6:**
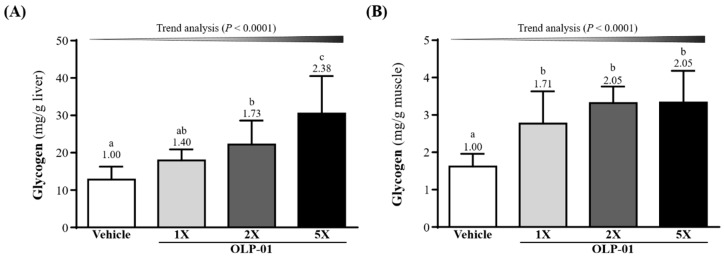
Effect of 4 week OLP-01 supplementation on (**A**) hepatic glycogen and (**B**) muscle glycogen levels. Data are expressed as mean ± SD for *n* = 10 mice per group. Values with different superscript letters (a, b, c) are significantly different at *p* < 0.05.

Glycogen stores in liver were elevated 1.73- (*p* = 0.0023) and 2.38-fold (*p* < 0.0001), respectively, in the OLP-01-2X and OLP-01-5X groups compared with vehicle group (Figure 6A). OLP-01 supplementation (OLP-01-1X, OLP-01-2X, and OLP-01-5X) significantly increased the muscle glycogen levels over the vehicle group 1.71-fold (*p* = 0.0004), 2.05-fold (*p* < 0.0001), and 2.05-fold (*p* < 0.0001), respectively (Figure 6B). A significant dose-dependent effect of OLP-01 on hepatic and muscular glycogen content was observed (*p* < 0.0001).

### 3.8. Effect of OLP-01 Supplementation on Biochemical Profiles at the End of the Study

We investigated whether the beneficial effects of OLP-01 supplementation on grip strength, exhaustive exercise challenge, and anti-fatigue performance led to changes in related biochemical parameters (Table 3). The liver damage markers AST and ALT were not significantly different between the groups (*p* > 0.05). Mean levels of other biochemical indices, including albumin, TC, TG, CK, BUN, creatinine, UA, TP, and glucose, were also found to be unchanged between the groups (*p* > 0.05). Our results suggest that the doses of OLP-01 supplementation used in the present study are safe, with little effect on biochemical indices.

### 3.9. Effect of OLP-01 Supplementation on the Gut Microbiota

We analyzed the gut microbiota composition using the 16S rRNA genes in the vehicle or OLP-01-treated mice, and observed dramatic changes in the microbial ecology when treated with OLP-01 at the end of experiment. To know whether the supplementation with OLP-01 had altered the gut microbiota, we chose the highest dose of OLP-01-5X to compare with the vehicle. As shown in Table 4, the genus counts of the gut microbiota of the mice on OLP-01-5X showed slightly increased *Lactobacillus* (205.66%), *Clostridium* (104.51%), *Bifidobacterium* (164.25%), and *Lactococcus* (571.76%), and simultaneously decreased *Bacillus* (92.34%), *Enterococcus* (73.95%), and *Akkermansia* (92.34%) compared to vehicle.

Since OLP-01 is a subspecies of *B. longum*, it was necessary to count the species of *Bifidobacterium.* The OLP-01-5X counts in any species of *Bifidobacterium* were higher than the vehicle (109–1202%); among then, the *B. longum* was slightly increased over vehicle by 1210% *(p* = 0.0693).

### 3.10. Effect of OLP-01 Supplementation on Tissue Histology at the End of the Study

**Figure 7:**
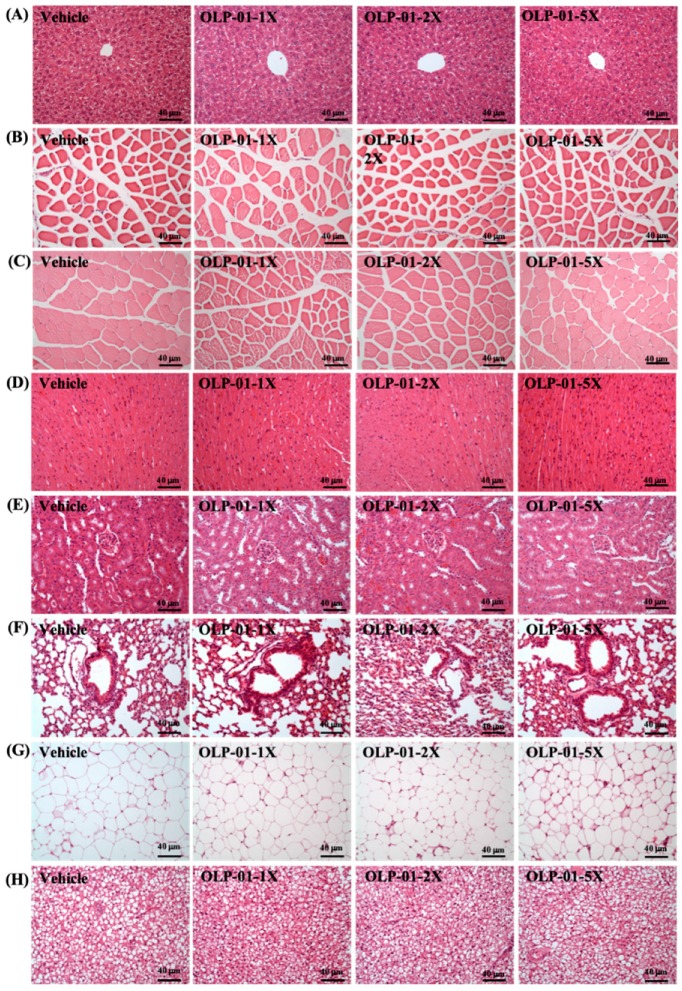
Effect of OLP-01 supplementation on histomorphology of the (**A**) liver, (**B**) muscle, (**C**) quadricep muscles, (**D**) heart, (**E**) kidney, (**F**) lung, (**G**) adipocyte tissue, and (**H**) BAT tissue in mice. Specimens were photographed under a light microscope (H&E stain, magnification: 200×; bar, 40 μm).

A histological examination of the liver, muscle, heart, kidney, lung, EFP, and BAT obtained from the mice in the different groups was performed at the end of the study. Figure 7 shows representative photomicrographs of organs from the vehicle and OLP-01-supplemented mouse groups. Hematoxylin and eosin (HE) stains of all the liver tissues revealed normal hepatic architecture of hepatocytes, bile duct, and sinusoid. Hypertrophy and hyperplasia were not observed in any of the heart cardiomyocytes or skeletal muscles. Renal sections also revealed normal renal architecture, with a normal appearance of glomeruli, tubules, and interstitial tissues. In addition, there was no difference in the morphology of adipose tissue or fat cell size between the groups. Taken together, the results suggest that OLP-01 supplementation at these doses was safe and did not lead to any adverse effects on major organs or tissues. We analyzed gut microbiota compositions using the 16S rRNA genes in the vehicle or OLP-01-supplementation-treated mice, and observed dramatic changes in the microbial ecology at the end of experiment when treated with OLP-01. To know whether the supplementation of OLP-01 bacteria had altered the intestinal flora, we chose the highest dose of OLP-01-5X to compare with the vector.

## 4. Discussion

In this study, we used *B. longum* (OLP-01) as a supplement to study its physiological effects on exercise performance and related parameters. We have shown that OLP-01 supplementation significantly promotes grip strength and exercise endurance performance without any training intervention. Biochemical indicators of fatigue and injury after exercise challenge, such as blood levels of lactate, BUN, NH_3_, and CK were found to be significantly reduced, while the recovery from exercise fatigue was accelerated with OLP-01 supplementation. This suggests a benefit of OLP-01 in improving physiological adaptation.

The effect of probiotics on exercise performance may vary according to the probiotic studied; different strains have different mechanisms of action, which have not been confirmed. Previous research has pointed out that prebiotic or probiotic supplementation could increase intestinal SCFA content [27,28], such as acetate, n-butyrate, and propionate. This could regulate host energy balance and increase nutrient availability [29]. In the case of *Bifidobacteria*, these monosaccharides are converted to intermediates of the hexose fermentation pathway, also known as the fructose-6-phosphate shunt or ‘bifid’ shunt [30,31]. They are ultimately converted to SCFAs and other organic compounds, some of which may be beneficial to the host. SCFAs are absorbed by intestinal mucosa, relatively high in caloric content, and metabolized by the colonocytes and hepatocytes. In colonocytes, butyrate is transported to mitochondria, where it undergoes fatty-acid oxidation (FAO) in aerobic conditions and becomes acetyl-CoA. The acetyl-CoA enters the Krebs cycle to produce NADH, which enters the electron transport chain and leads to adenosine triphosphate (ATP) and CO_2_ production [32]. Interestingly, n-butyrate could play an important role in peroxisome proliferator-activated receptor gamma coactivator-1α (PGC-1α) activation; it has been shown to dramatically increase in skeletal muscles in response to exercise [33], and induction leads to a transformation of skeletal muscle fibers from type II (glycolytic) to type I (oxidative). These type I fibers are rich in mitochondria and stimulate FAO for ATP production [34]. Therefore, it is possible that *PGC-1α* gene expression may be considered a potential biomarker of mitochondrial function during endurance training.

Forelimb grip strength is a physical test that reflects the overall health of the musculoskeletal system and can be used to evaluate motor-associated coordination and adaptation in neurological studies [35]. In this study, although the muscle mass of the OLP-01 and vehicle groups did not differ with 4 weeks of supplementation (Table 1), the forelimb grip strength was shown to increase significantly in the OLP-01 groups (Figure 2). In general, programmed exercise training is required to improve endurance capacity and grip strength [36]. Although no training program was implemented in this study, our experimental results indicate that endurance capacity may be improved and exercise-induced physical fatigue ameliorated by 4 weeks of OLP-01 supplementation (Figure 3).

Exercise related indexes like lactate, BUN, CK, and glucose are widely used to assess exercise physiological status. The energy metabolism pathway contains the phosphagen system, glycolysis, and the aerobic system, which are dependent on exercise intensity and duration for initiation [25]. Blood lactate is the glycolysis product of carbohydrate under anaerobic conditions, when glycolysis is the main energy source [37]. Hydrogen ions are produced together with lactate, probably due to the dissociation of lactic acid. A decrease in pH in blood and muscle tissue leads to inhibition of muscle contraction and glycolysis, as well as various deleterious biochemical, metabolic, and physiological side effects [38]. However, previous studies have pointed out that probiotics can improve the production of lactic acid during exercise and convert it into SCFAs, especially propionic acid and butyrate, and provide the energy needed for muscles during exercise [39]. We have shown that OLP-01 supplementation could significantly decrease exercise-induced lactate levels and improve lactate production rate (Table 2). Therefore, we suggest that the OLP-01 supplement is beneficial for the removal and utilization of lactic acid, thereby reducing the development of fatigue.

Another important indicator of fatigue is blood ammonia. During exercise, ammonia is produced and accumulates in skeletal muscle when adenosine monophosphate (AMP) is deaminated to inosine monophosphate (IMP) by AMP deaminase (AMPD) during resynthesis of ATP [26]. The ingestion of *Bifidobacterium* together with lactulose has been shown to assist in reestablishing the balance of the gut flora. This was accompanied by a decrease in fecal pH and reduction of ammonia and free phenols in the blood [40]. As seen in Figure 4A, supplementing mice with 4 weeks of OLP-01 effectively reduced blood ammonia concentration after exercise. Ammonia may be metabolized as BUN via the urea cycle [41], thus BUN could be considered a biomarker for ATP metabolism, and not just a marker for kidney function. After the 90 min swim test and 60 min rest period, BUN was shown to be significantly decreased in the OLP-01-supplemented mice (Figure 5A).

Glucose is stored as glycogen, which is an important energy source for ATP production and is mainly present in the liver and muscle tissues. Glycogen is able to complement the consumption of blood glucose during exercise and helps to maintain blood glucose levels within the physiological range [42]. There is a high correlation between glycogen storage and carbohydrate uptake and metabolism [43]. Previous studies have shown that *B. longum* is able to transport a variety of disaccharides and oligosaccharides, e.g., oligofructose, which are described as growth-promoting prebiotics. More than half of the transport systems are ATP-dependent ATP-binding cassette (ABC)-type permeases. *B. longum* has a glucose-specific phosphoenolpyruvate-dependent sugar phosphotransferase system (PTS), while two other *Bifidobacterium* species (*lactis* and *bifidum*) have a fructose-6-phosphate-forming fructose-PTS instead. It has become obvious that apart from a few exceptions, most carbohydrate systems are closely related to those from other actinomycetes [44]. In this study, supplementation with OLP-01 effectively increased liver and muscle glycogen storage (Figure 6A,B), improving glycogen degradation and exercise performance.

Probiotics may be used as a supplement to meet most of our basic nutritional and health requirements. Previous studies have shown *B. longum* to be a safe bacteria strain [11]. We also observed the safety of *B. longum* in 4 weeks of OLP-01 supplementation in the mice. Serum metabolic markers, including liver and renal function tests, were found to be within the normal range, even after several exercise challenges. In pathological sections, we also did not detect any gross abnormalities or obvious lesions in the various tissues and organs. Taken together, we believe that the supplementation of OLP-01 (*B. longum)* improve gastrointestinal symptoms, accelerate recovery fatigue, and improve exercise performance, and should not lead to any safety concerns.

## 5. Conclusions

In the current study, we found that four weeks’ supplementation of OLP-01, a bacterial strain isolated from a top weightlifting athlete, could increase liver glycogen level and improve both grip strength and endurance in mice. In addition, OLP-01 showed anti-fatigue properties by lowering serum lactate, ammonia, and CK levels in a dosage-dependent manner. We suggest that OLP-01 could be used as a supplement to modify gut microbiota and enhance exercise performance while mitigating fatigue. The mechanism and pathway activation by OLP-01 warrants further investigation.

## Figures and Tables

**Table 1:** General characteristics of the experimental groups.

Characteristic	Vehicle	OLP-01-1X	OLP-01-2X	OLP-01-5X	Trend Analysis
Initial BW (g)	30.6 ± 0.7	30.3 ± 0.6	30.5 ± 1.6	30.9 ± 2.1	0.4253
Final BW (g)	38.5 ± 2.3	38.1 ± 2.7	38.8 ± 2.9	38.8 ± 3.2	0.5491
Water intake (mL/mouse/day)	7.4 ± 0.7	7.5 ± 0.8	7.4 ± 1.0	7.4 ± 1.4	0.3575
Diet intake (g/mouse/day)	7.9 ± 1.1	7.9 ± 1.0	7.8 ± 1.8	7.9 ± 1.5	0.7006
Liver (g)	2.16 ± 0.15	2.19 ± 0.27	2.22 ± 0.16	2.22 ± 0.25	0.5576
Muscle (g)	0.39 ± 0.03	0.39 ± 0.03	0.39 ± 0.03	0.39 ± 0.05	0.8603
Quadriceps (g)	0.51 ± 0.05	0.51 ± 0.04	0.52 ± 0.04	0.51 ± 0.05	0.8678
Kidney (g)	0.70 ± 0.09	0.70 ± 0.08	0.71 ± 0.12	0.71 ± 0.09	0.3795
Heart (g)	0.20 ± 0.03	0.20± 0.03	0.20 ± 0.02	0.20 ± 0.02	0.7699
Lung (g)	0.23 ± 0.03	0.24 ± 0.03	0.23 ± 0.03	0.24 ± 0.03	0.8482
EFP (g)	0.38 ± 0.16	0.29 ± 0.10	0.29 ± 0.09	0.29 ± 0.07	0.3066
BAT (g)	0.08 ± 0.01	0.08 ± 0.01	0.09 ± 0.03	0.09 ± 0.01	0.2921
Cecum (g)	0.79 ± 0.20	0.77 ± 0.05	0.78 ± 0.10	0.79 ± 0.16	0.5076
Relative liver weight (%)	5.62 ± 0.15	5.73 ± 0.32	5.72 ± 0.12	5.71 ± 0.23	0.2903
Relative muscle weight (%)	1.01 ± 0.04	1.01 ± 0.03	1.00 ± 0.02	1.01 ± 0.05	0.6849
Relative quadriceps weight (%)	1.33 ± 0.07	1.34 ± 0.03	1.33 ± 0.06	1.33 ± 0.03	0.0896
Relative kidney weight (%)	1.80 ± 0.14	1.83 ± 0.12	1.83 ± 0.22	1.83 ± 0.10	0.7062
Relative heart weight (%)	0.52 ± 0.04	0.52 ± 0.04	0.50 ± 0.03	0.51 ± 0.03	0.4263
Relative lung weight (%)	0.60 ± 0.05	0.62 ± 0.04	0.60 ± 0.03	0.61 ± 0.03	0.8376
Relative EFP weight (%)	0.97 ± 0.36 ^b^	0.74 ± 0.21 ^a^	0.73 ± 0.18 ^a^	0.74 ± 0.12 ^a^	0.1584
Relative BAT weight (%)	0.22 ± 0.03	0.20 ± 0.01	0.22 ± 0.05	0.23 ± 0.02	0.5144
Relative cecum weight (%)	2.02 ± 0.42	2.01 ± 0.30	2.00 ± 0.12	2.03 ± 0.28	0.9822

Data are expressed as mean ± SD for *n* = 10 mice in each group. Data in the same row with different letters (a, b) differ significantly at *p* < 0.05 by one-way ANOVA; EFP: epididymal fat pad; BAT: brown adipose tissue.

**Table 2:** The effect of OLP-01 on lactate levels during acute exercise challenge.

Time Point	Vehicle	OLP1-01-1X	OLP-01-2X	OLP-01-5X	Trend Analysis
	Lactate (mmol/L)				
Before swimming (A)	3.42 ± 0.37	3.43 ± 0.36	3.41 ± 0.30	3.43 ± 0.24	0.7111
After swimming (B)	6.74 ± 0.28 ^b^	5.64 ± 0.57 ^a^	5.40 ± 0.83 ^a^	5.12 ± 0.59 ^a^	<0.0001
After a 20 min rest(C)	6.05 ± 0.25 ^b^	5.04 ± 0.65 ^a^	4.76 ± 0.67 ^a^	4.53 ± 0.57 ^a^	<0.0001
Rate of lactate production and clearance					
Production rate = B/A	1.99 ± 0.23 ^c^	1.64 ± 0.05 ^b^	1.58 ± 0.12 ^ab^	1.49 ± 0.10 ^a^	<0.0001
Clearance rate = (B − C)/B	0.10 ± 0.02	0.11 ± 0.06	0.12 ± 0.03	0.12 ± 0.02	0.0222

The metabolite lactate was assessed for the four groups: vehicle, OLP-01-1X, OLP-01-2X, and OLP-01-5X at three time points. Lactate production rate (B/A) was calculated as the lactate level after exercise (B) divided by the lactate level before exercise (A). Clearance rate at rest (B − C)/B was defined as the difference in lactate levels immediately after exercise (B) and after rest (C), divided by lactate level after rest (B). Data are expressed as mean ± SD for *n* = 10 mice per group. Values in the same row with different superscript letters (a, b, c) differ significantly, *p* < 0.05.

**Table 3:** The effects of OLP-01 on biochemical indices at the end of the study.

Parameter	Vehicle	OLP-01-1X	OLP-01-2X	OLP-01-5X	Trend Analysis
AST (U/L)	68 ± 11	68 ± 10	69 ± 27	68 ± 13	0.9451
ALT (U/L)	31 ± 8	32 ± 10	31 ± 15	32 ± 8	0.4689
CK (U/L)	162 ± 52	129 ± 65	120 ± 69	128 ± 94	0.0751
GLU (mg/dL)	229 ± 21	231 ± 48	227 ± 24	228 ± 26	0.8925
CREA (mg/dL)	0.38 ±0.02	0.37 ± 0.02	0.37 ± 0.03	0.38 ± 0.02	0.9674
BUN (mg/dL)	23.4 ± 1.6	23.4 ± 1.7	23.7 ± 2.8	22.3 ± 1.4	0.8665
UA (mg/dL)	2.6 ± 0.6	2.5 ± 0.8	2.5 ± 0.5	2.5 ± 0.3	0.8331
TC (mg/dL)	138 ± 32	133 ± 24	135 ± 15	135± 13	0.9813
TG (mg/dL)	141 ± 27	141 ± 13	141 ± 20	142 ± 13	0.5683
ALB (g/dL)	3.0 ± 0.1	3.0 ± 0.2	3.0 ± 0.2	3.1 ± 0.1	0.0915
TP (g/dL)	5.3 ± 0.2	5.4 ± 0.2	5.4 ± 0.3	5.4 ± 0.2	0.0630

Data are expressed as mean ± SD for *n* = 10 mice in each group. AST, aspartate aminotransferase; ALT, alanine transaminase; CK, creatine kinase; GLU, glucose; CREA, creatinine; BUN, blood urea nitrogen; UA, uric acid; TC, total cholesterol; TG, triacylglycerol; ALB, albumin; TP, total protein.

**Table 4:** The effects of OLP-01 on the gut microbiota at the end of the study.

Counts	Vehicle	OLP-01-5X	*p* Value	Compared with Vehicle (%)
**Genus**				
*Lactobacillus*	842.36 ± 116.85	1732.35 ± 1290.13	0.2185	205.66
*Clostridium*	1028.05 ± 349.22	1074.37 ± 190.07	0.8235	104.51
*Bacillus*	581.67 ± 174.5	537.12 ± 94.68	0.6693	92.34
*Enterococcus*	179.41 ± 105.22	132.67 ± 59.86	0.4693	73.95
*Bifidobacterium*	55.05 ± 7.85	90.42 ± 30.04	0.0630	164.25
*Streptococcus*	55.5 ± 10.55	55.25 ± 15.36	0.9796	99.55
*Akkermansia*	68.07 ± 47.44	27.07 ± 9.71	0.1413	39.77
*Lactococcus*	0.23 ± 0.45	1.3 ± 1.69	0.2662	571.76
**Species**				
*Bifidobacterium bombi*	13.85 ± 4.68	21.22 ± 10.27	0.2392	153.23
*Bifidobacterium longum*	1.25 ± 1.72	15.11 ± 12.43	0.0693	1210.56
*Bifidobacterium asteroides*	3.15 ± 1.70	3.84 ± 1.50	0.5626	122.05
*Bifidobacterium indicum*	2.51 ± 1.36	2.71 ± 1.18	0.8295	108.06
*Bifidobacterium catenulatum*	0 ± 0	6.22 ± 2.75	0.0040 *	-
*Bifidobacterium scardovii*	0.63 ± 0.56	1.89 ± 2.22	0.3128	298.82
*Bifidobacterium stercoris*	0.84 ± 1.69	1.66 ± 1.87	0.5412	196.58
*Bifidobacterium choerinum*	0.77 ± 0.87	1.08 ± 0.55	0.5653	140.76
*Bifidobacterium subtile*	0.16 ± 0.33	1.8 ± 2.19	0.1902	1092.70
*Bifidobacterium adolescentis*	0.17 ± 0.34	0.95 ± 1.36	0.3060	563.98
*Bifidobacterium kashiwanohense*	0.13 ± 0.26	0.38 ± 0.46	0.3859	288.94
*Bifidobacterium bifidum*	0 ± 0	1.26 ± 2.53	0.3559	-
*Bifidobacterium cuniculi*	0.84 ± 1.69	0.21 ± 0.42	0.4938	24.93
*Bifidobacterium merycicum*	0 ± 0	0.12 ± 0.23	0.3559	-
*Bifidobacterium magnum*	0.13 ± 0.27	0 ± 0	0.3559	0.00

Data are expressed as mean ± SD for *n* = 10 mice in each group. Values in the same row with different superscript letters (*) differ significantly, *p* < 0.05, by t test.
